# Validating a Self-Report Measure of Student Athletes’ Perceived Stress Reactivity: Associations With Heart-Rate Variability and Stress Appraisals

**DOI:** 10.3389/fpsyg.2019.01083

**Published:** 2019-05-09

**Authors:** Darren M. Britton, Emma J. Kavanagh, Remco C. J. Polman

**Affiliations:** ^1^School of Sport, Health, and Social Sciences, Solent University, Southampton, United Kingdom; ^2^Department of Sport and Physical Activity, Bournemouth University, Bournemouth, United Kingdom; ^3^School of Exercise and Nutrition Sciences, Queensland University of Technology, Brisbane, QLD, Australia

**Keywords:** stress reactivity, sport, adolescence, heart-rate variability, stress appraisals, cold pressor test

## Abstract

Student athletes experience multiple stressors relating to both their sporting and academic commitments. Individual differences play a significant role in how well student athletes cope with the demands they face. When assessing individual differences in stress reactivity, there are a lack of valid alternatives to costly and time-consuming lab-based physiological methods (e.g., cortisol sampling, cardiac variables). This paper aims to further validate a self-report measure of adolescent athletes’ individual differences in perceived stress reactivity, by comparing to a psycho-physiological measure of emotion regulation (heart-rate variability) assessed during a socially evaluated cold pressor test. 30 student athletes and 31 student non-athletes completed a measure of perceived stress reactivity and took part in the socially evaluated cold pressor test while their heart-rate variability was assessed, along with their self-reported appraisals of stress, pain, and unpleasantness experienced during the procedure. Controlling for gender and athleticism, individual differences in perceived stress reactivity showed no associations with tonic or phasic levels of heart-rate variability. However, perceived stress reactivity was associated with levels of self-reported stress, pain, and unpleasantness experienced during the socially evaluated cold pressor test. These findings therefore suggest that perceived stress reactivity is associated with cognitive responses to stress (i.e., stress appraisals). However, further research is needed to confirm its relationship with physiological measures and responses. This further adds to the understanding of perceived stress reactivity, and validity of the perceived stress reactivity scale for adolescent athletes.

## Introduction

Adolescent athletes experience a significant number of stressors when taking part in competitive sport (Reeves et al., 2011; Hayward et al., 2017). These can range from performance related stressors, such as physical and mental errors, as well as organizational stressors, such as conflicts with team-mates and coaches (Arnold et al., 2017). There are also multiple stressors associated directly with adolescence, such as academic commitments and peer and family relationships (Compas et al., 2001; van Rens et al., 2016). In addition, an adolescent’s sensitivity to stress is heightened during adolescence, due to their stage of neurological and physiological development (Romeo, 2010; Ahmed et al., 2015). These developments continue well into an individual’s mid-twenties (Blakemore and Choudhury, 2006). Therefore, recent research has recommended expanding the traditional age brackets of adolescence to 10 to 25 years of age in order to reflect this (Sawyer et al., 2018). In other words, an individual is still arguably an “adolescent" until they are 25 years of age. An inability to cope adaptively with stressors has been associated with increased levels of burn-out and drop-out in youth sport and attributed to talented youth athletes’ struggling to succeed later in their careers at adult level (Holt and Dunn, 2004; Goodger et al., 2007; Crane and Temple, 2015).

Lazarus and Folkman (1987) proposed that stress emerges as an interaction between an individual and their environment, plus the subjective appraisal of stressful events. Therefore, numerous individual differences have been shown to influence how adolescent athletes appraise and cope with stress, including gender (Nicholls et al., 2007; Kaiseler et al., 2012b), the Big Five personality traits (Kaiseler et al., 2012a), and mental toughness (Kaiseler et al., 2009). For example, athletes measuring higher in the trait of neuroticism are more likely to appraise stressors with greater perceived intensity (Kaiseler et al., 2012a). The physical, emotional, and cognitive-social maturity of adolescent athletes have also been shown to influence how they cope with stress (Nicholls et al., 2009, 2013, 2015). Given the increased sensitivity to stress experienced by adolescents during this period, little research has examined the effects of individual differences in stress reactivity on the performance and well-being of adolescent athletes. Given the vast number of stressors which young athletes encounter during the vulnerable developmental period of adolescence, increasing the risk of burnout and dropout from sport, a better understanding of the mechanisms and effects of stress reactivity among adolescent athletes is important.

### Stress Reactivity

Personality is ultimately the result of differential reactivity to stimulation from the environment, with introversion and neuroticism being the result of hyper-reactivity (Suls and Martin, 2005). Stress reactivity (SR) has been identified as an individual difference that underlies this broad variability in responses to stress (Boyce and Ellis, 2005; Ellis et al., 2005; Schlotz, 2013). SR develops during adolescence via a period of increased sensitivity (Romeo, 2010; Dumontheil, 2016). A recent meta-analysis has also identified that adverse childhood experiences (such as physical, sexual, and emotional abuse, neglect, and poverty) also contribute to the development of increased SR in later life (Hughes et al., 2017). Hyper-reactivity in adolescents has been associated with internalizing symptoms during adolescence (negative emotionality, anxiety, and depression; Granger et al., 1994; Allwood et al., 2011; Lopez-Duran et al., 2015). In adolescent athletes, self-reported SR has been associated with reduced satisfaction across multiple life domains, and greater levels of perceived stress over time (Britton et al., 2017). Measuring individual differences in adolescent athletes’ SR allows both researchers and practitioners to identify young performers at greatest risk of experiencing negative symptoms and adverse outcomes. Furthermore, the development of reliable measures of SR would facilitate further research regarding how athletes develop during adolescence.

In sporting contexts, it has been considered difficult to reliably measure SR as a stable individual difference (Polman et al., 2010). Adolescent SR is typically measured using physiological or neuroendocrine measures in controlled lab settings (Allwood et al., 2011; Marceau et al., 2012; McLaughlin et al., 2014; Colich et al., 2015; Paysnick and Burt, 2015). However, sporting environments are dynamic by nature, with numerous environmental and situational factors influencing stress responses. It is also difficult to delineate between physiological arousal as a consequence of SR, and physiological arousal in response to the physical demands of sport (Polman et al., 2010). Lab procedures adopted to measure reactivity are also often costly, time-consuming, invasive, and lack ecological validity (Schlotz et al., 2011a).

### Perceived Stress Reactivity

Britton et al. (2017) developed the Perceived Stress Reactivity Scale for Adolescent Athletes (PSRS-AA). This was adapted from an existing scale (Schlotz et al., 2011b), in order to specifically assess adolescent athletes’ perceived stress reactivity (PSR) across a number of potentially stressful situations. The original PSRS reflects reactivity to a number of potential stressful situations (e.g., “when I have to speak in front of other people”), while the PSRS-AA was re-worded to reflect sport specific situations of a similar nature (e.g., “when I have to perform in front of other people”). The construct of PSR has been defined as a disposition that underlies individual differences in physiological and psychological responses to stress (Schlotz et al., 2011b). PSR is particularly pertinent to Lazarus and Folkman’s (1987) transactional model of stress, given that physiological and psychological responses to stress are the result subjective appraisals. However, there have been mixed and equivocal findings with regards to the relationship between measures of perceived reactivity and physiological and neuroendocrine indexes of SR (Schlotz et al., 2011a; Evans et al., 2013).

Schlotz et al. (2011b) found scores on the original PSRS’s reactivity to social evaluation (RSE) subscale to be associated with elevated cortisol responses to a Trier Social Stress Test. However, this relationship was mediated by the primary appraisal of threat. Evans et al. (2013) examined the association between a number of physiological and neuroendocrine indices and perceived reactivity in adolescents. Only cortisol reactivity (not heart-rate or respiratory sinus arrhythmia) was associated with adolescents’ perceptions on their physiological responses Therefore, more research is required to establish whether self-report measures such as the PSRS-AA are valid alternatives to lab-based measures, particularly physiological indices.

The PSRS-AA was initially validated by Britton et al. (2017). Confirmatory factor analysis demonstrated an adequate second order model fit for a five-factor structure reflecting reactivity to five different stress domains (RSE, reactivity to social conflict, reactivity to failure, reactivity to work overload, and prolonged reactivity) and an overall aggregate score of total reactivity. This aggregate score was found to have good internal consistency and test retest reliability. Support for its criterion validity includes associations with specific personality traits (high neuroticism, high introversion, and low openness), reduced life satisfaction across multiple domains, and higher levels of perceived stress over time (Britton et al., 2017).

Although initial validity for the scale has been provided by Britton et al. (2017), further research is required to build the evidence base surrounding the PSRS-AA. This is in relation to its ability to predict physiological stress responses, and thus its validity as an alternative measure. Therefore, the following study aimed to achieve this, in part, by examining the criterion validity of the PSRS-AA in relation to several other measures and related constructs.

### Emotion Regulation and Heart-Rate Variability

The processes of both stress-coping and emotion regulation share many similarities and differences (Wang and Saudino, 2011). Stress-coping involves consciously changing efforts to manage internal or external demands. Emotion regulation, on the other hand, can involve both implicit and explicit modulation of internal emotional changes to meet demands (Gross and Jazaieri, 2014). Furthermore, emotion regulation does not always occur in response to a specific situation or event and involves the control and modulation of both positive and negative emotions (Wang and Saudino, 2011). Despite these differences, stress-coping and emotion regulation share many of the same neural networks. Specifically, they are both associated with activation of the pre-frontal cortex (PFC), and modulation of the amygdala. Furthermore, both processes are associated with activation of the hypothalamic-pituitary-adrenal axis (HPA). Emotion regulation predicts elevations in cortisol in response to stress, and both processes involve the modulation of both affect and appraisals of events or states (Wang and Saudino, 2011). Of importance to this study, the shared neural networks and structures associated with both stress-coping and emotion regulation develop during adolescence and into young adulthood (Blakemore and Choudhury, 2006; Ahmed et al., 2015). Therefore, one would propose that measures of adolescent athletes’ PSR are likely to be related to measures of emotion regulation.

High-frequency heart rate variability (HF-HRV; 0.15–0.40 Hz) can be used as physiological index of emotion regulation (Thayer et al., 2012). The Neurovisceral Integration model proposes an adaptive system of neural structures that regulate cognition, perception, action, and physiology in the face of physiological and environmental demands. This includes a bi-directional connection between the heart and the brain via the vagus nerve. Higher tonic levels (HF-HRV over a given period) and greater phasic increases (changes in HF-HRV across periods) in HF-HRV index activation of the parasympathetic nervous system, and thus the PFC. This in turn increases the PFC’s inhibitory control over the amygdala; regulating emotions. Predispositions to high levels of SR are likely to dysregulate this integrative system, with greater sympathetic activation, decreased activation of the PFC, followed by disinhibition of the amygdala, indexed by low levels of HF-HRV (Thayer et al., 2012).

Heart rate variability can also be indexed using time-domain indices (such as the root mean square of successive differences; RMSSD), which reflects beat to beat variance in heart rate (Shaffer and Ginsberg, 2017). However, for the present study HF-HRV derived from frequency domain measurements were examined exclusively for two reasons. Firstly, HF-HRV provides a greater reflection of the HR variations related to the respiratory cycle. Secondly, HF-HRV is strongly correlated with time-domain measures such as RMSSD (Shaffer and Ginsberg, 2017). Thirdly, previous studies with athletes have exclusively measured HF-HRV and not time-domain indices (Laborde et al., 2015; Mosley et al., 2017).

Gender differences have been observed in HF-HRV, with a meta-analysis suggesting that women experience higher levels of HF-HRV than men (Koenig and Thayer, 2016). Greater levels aerobic fitness has been shown to influence faster recoveries in HRV after exercise (Stanley et al., 2013). Furthermore, recent research has identified associations between HF-HRV and performance related variables under pressure conditions (Laborde et al., 2015; Mosley et al., 2017). Therefore, one would expect that, controlling for gender and athleticism, PSRS-AA scores would be associated with tonic and phasic measurement of HF-HRV in response to a novel stressor featuring physical and/or socially evaluative threat. This would also support that the processes of stress and emotion regulation are related. Furthermore, it would further support the use of the PSRS-AA as a valid alternative to lab-based methods of indexing individual differences in SR.

### Aims and Hypotheses

The aim of this study was to examine the validity of the RSE subscale of the PSRS-AA in relation to a physiological measure of emotion regulation: HF-HRV. This would enhance the validity of the PSRS-AA as a self-report alternative to psycho-physiological measures of individual differences in SR. The study also examined the validity of a socially evaluated cold pressor test (SECPT; Schwabe et al., 2008) for use with adolescent athletes, and to examine gender and athleticism differences in response to the SECPT in student athletes and non-athletes. This was due to previous research which has found HF-HRV differences between genders and levels of athletic fitness (Stanley et al., 2013; Woo and Kim, 2015). This study also aimed to examine whether these differences could be observed using the SECPT.

The combination of both HF-HRV and the SECPT were used for the following reasons. Firstly, a reduction in HF-HRV would indicate vagal withdrawal and sympathetic dominance, thus reflecting increased stress reactivity (Shaffer et al., 2014). Secondly, abrupt changes in temperature provoke vagal withdrawal over short periods (Peng et al., 2015). Therefore, with the SECPT only featuring an immersion length of 3 min, along with its socially evaluative elements, a decrease in HF-HRV would be expected.

It was predicted that: controlling for gender and athleticism, the RSE subscale would relate to tonic levels and phasic changes in HF-HRV before, during, and after the SECPT. Specifically, higher levels of self-reported RSE would be associated with greater decreases in HF-HRV from before the task to during (reactivity), and smaller increases in HF-HRV after the task (recovery; Laborde et al., 2017); finally, also controlling for gender and athleticism, the RSE subscale would also be associated with greater ratings of perceived stress, pain, and unpleasantness immediately after the task.

In order to confirm the validity of the SECPT as a method of stress induction for student athletes and non-athletes, a number of additional predictions were made: There would be significant changes in HF-HRV from before to during the task, with reactivity being indexed by a reduction in HF-HRV, recovery by an increase; lower levels of HF-HRV reactivity during the task would be associated with subjective appraisals of greater stress, pain, and unpleasantness immediately after the task; greater levels of tonic and phasic HF-HRV would be observed in females; greater levels of tonic and phasic HF-HRV would be observed in student athletes compared to student non-athletes.

## Materials and Methods

### Participants

Sixty-one students were recruited to the study (*M* Age = 20.11, *SD* = 1.25, Male *N* = 28, Female *N* = 33). 30 Of the students participated in a wide range of competitive sports at various levels, including football, weightlifting, athletics, sailing, swimming, rugby union, golf, climbing, martial arts, tennis, and netball. 31 students did not take part in any competitive sport at any level. All participants were in full-time education at a United Kingdom university. The study was approved by a university ethics committee and all participants provided written consent prior to testing.

### Measures

The RSE subscales of the PSRS-AA (for student athletes) and the original PSRS (for student non-athletes) was used for this study. This was due to the conditions of social evaluation threat the SECPT is designed to produce. Although previous research has demonstrated questionable reliability of the PSRS-AAs RSE subscale (α = 0.66; Britton et al., 2017), data collected from this study produced good reliability for the subscale (α = 0.78).

Heart rate variability was measured using the eMotion Faros 180° device (Mega Electronics Ltd., Pioneerinkatu, Finland). Two pre-lubricated disposable electrodes (Ambu VLC-00-S/25, Ambu GmbH, Bad Nauheim, Germany) were placed below the left clavicle and the left side below the 12th rib. Immediately after completing the SECPT, participants rated their perceptions of how stressful, painful, and unpleasant the task was, on three visual analog scales (VAS) of 10 centimeters in length, from 0 (“not at all”) to 10 (“very much”).

### Procedure (SECPT)

Prior to testing, participants completed the PSRS-AA or PSRS electronically, and were invited via email to take part in the SECPT. Participants were asked to refrain from eating, consuming caffeine, smoking within 2 h of testing, and consuming alcohol within 24 h of testing, due to their impact on cardiac variables (Laborde et al., 2017). Participants also had to avoid any intensive physical activity within 24 h of testing and to have adhered to their regular sleeping pattern. Participants were asked if they had any cardiac disease, respiratory disorder, or blood pressure problems, or were taking any medication that may affect the heart and thus the results of the study. Using a pre-testing checklist based upon recommendation by Laborde et al. (2017), none of the above were reported prior to testing. Water consumption, however, was not controlled for prior to testing.

On arrival, participants were informed that they would be video-taped while they performed the task. This was to achieve the social evaluative element of the SECPT. Participants then provided written informed consent before checking they had adhered to the pre-testing requirements. Participants were then fitted with the Faros 180° device, seated in a chair, and asked to remain as still as possible while resting HF-HRV was measured for 3 min. This was to achieve a baseline measure of HF-HRV at rest. The SECPT uses 3-min intervals for pre, task, and post-task (Schwabe et al., 2008). The video camera positioned directly in front of them three meters away was then turned on. After the completion of the baseline measure, participants were instructed to submerge their right hand, up to and including their wrist, into an insulated box of cold ice water (0–4°C) positioned on a platform directly next to them, while remaining as still as possible (to not disrupt the measurement of HF-HRV) and maintaining eye contact with the camera lens. They were encouraged to keep their hand in the water for up to a maximum of 3 min, however, they were informed that they had the right to remove their hand from the water at any time if they felt that they could no longer keep it submerged, and that this would not affect the results. During the SECPT the primary researcher sat in an observer’s position directly next to the camera, facing the participant. Upon completion of the task (either after 3 min or on voluntary termination), the camera was switched off, and participants were asked to complete the VAS scales of stressfulness, pain, and unpleasantness. The time at which participants withdrew their hand from the water was noted before completing the VAS scales and was recorded again before beginning the post-task rest period. This was to exclude the potential interference of the completing of the VAS scales from the analysis of data in Kubios. Participants were then asked to remain still and seated for a further 3 min to assess HF-HRV during recovery post-task. The Faros 180° device was then removed, and the data saved, while participants were debriefed.

### Data Preparation

High-frequency heart rate variability data from two participants was unable to be analyzed due to equipment failure. Furthermore, two participants’ post-task data was unable to be analyzed. Finally, one participant withdrew from the study before completing the task, having had their HF-HRV measured at rest. The HF-HRV data was processed for artifacts using Kubios. The low threshold was applied, and the data was visually checked. If artifacts were identified, artifact correction was applied at the low threshold level.

Absolute power statistic was used, derived from the autoregressive (AR) spectrum (between 0.15 and 0.40 Hz), which is deemed a reliable measure of HF-HRV and parasympathetic activation (Laborde et al., 2017). Raw data was compared to normative HF-HRV data (Shaffer and Ginsberg, 2017) to identify potential univariate outliers. Three cases were removed as they were much higher than expected based upon normative values. A log10 transform was applied on the HF-HRV data as it was not normally distributed. This is common practice with data obtained from the absolute power statistic (Laborde et al., 2017). Once this was performed, the data was visually checked and deemed to be normally distributed.

Indicators of reactivity (the phasic change between rest and task levels, achieved by subtracting pre-task HF-HRV from task HF-HRV) and recovery (the phasic change between task and post-task levels, achieved by subtracting task HF-HRV from post-task HF-HRV) were then calculated. PSRS-AA and VAS score data was visually checked for normality via histograms and boxplots.

### Data Analysis

An *a priori* analysis was initially performed to calculate the required sample size using Gpower v3.1. Due to medium effect sizes being reported in previous studies (Laborde et al., 2015; Mosley et al., 2017), a medium effect size (*f*^2^ = 0.15), α error probability of >0.05, and power (1-β error probability) of 0.8 were input as parameters, resulting in a minimum required sample size of *N* = 55. This minimum requirement was met with the recruitment of 61 participants. To assess the effectiveness of the SECPT in inducing stress responses in the sample, a set of analyses were performed.

Firstly, paired samples *t*-tests were performed between the tonic measurements of HF-HRV measured before and during the task, and during and after the task. This was to establish if there were significant differences between the tonic measures of HF-HRV at different stages of the SECPT. Secondly, Pearson’s r correlations were calculated between the measures of HF-HRV (tonic measure during the task, and phasic measure of reactivity from rest to task) and the VAS scales of stressfulness, pain, and unpleasantness (*r* correlations from 0.10 to 0.29 classified as small, 0.30 to 0.49 as medium, and 0.50 and above as large). This was to establish whether tonic and phasic measures of HF-HRV were correspondingly related to the participant’s subjective appraisals of stress during the task. Based on the visual check of histograms, and skewness and kurtosis values, the VAS measures were observed to be normally distributed.

To further examine potential differences in HF-HRV in relation to gender and athleticism, a two-way MANOVA was performed. Independent factors were gender and whether the participants were involved in competitive sport (athleticism), while all five measures of tonic and phasic HF-HRV were examined as dependent variables. Given the unequal group sizes for both gender and athleticism, Pillai’s trace was used to assess within-subjects effects. Effect sizes were determined using partial η^2^ (0.01 = small; 0.06 = medium; large = 0.14; Cohen, 1988).

To explore the validity of the RSE subscale, multiple regression analyses using the enter method were performed. Gender and athleticism were controlled for at step one, before entering the RSE subscale at step two. B, beta, adjusted R^2^, and ΔR^2^ were all included in the output. Analyses were performed for all five measures of tonic and phasic HF-HRV, and for the three VAS measures of self-reported stress, pain, and unpleasantness.

## Results

### SECPT Validity

Table 1 shows means and standard deviations for the HF-HRV and VAS measures. Paired samples *t*-tests revealed no significant differences between HF-HRV from rest to task: *t*(57) = 0.85, *p* = 0.40. Furthermore, there were no significant differences between HF-HRV from task to post-task: *t*(55) = -1.17, *p* = 0.25. Table 2 details Pearson’s r correlations HF-HRV variables (during task and reactivity) and VAS measures of subjective stressfulness, pain, and unpleasantness. Medium significant negative correlations were observed between HF-HRV during the task and perceived stressfulness, perceived pain, and perceived unpleasantness. No significant correlations were observed between HF-HRV reactivity and any of the subjective VAS measures.

**Table 1:** Means and standard deviations for HF-HRV variables, VAS scores, RSE subscale, and HR.

Variable	Group	Mean	SD
HF-HRV rest (Raw)	Athlete	1749.14	1443.93
	Non-athlete	1315.15	1317.19
HF-HRV task (Raw)	Athlete	1578.86	1512.10
	Non-athlete	1285.56	1203.26
HF-HRV post-task (Raw)	Athlete	1924.81	1728.29
	Non-athlete	1940.51	1755.47
HF-HRV reactivity (Raw)	Athlete	-6.38	1254.37
	Non-athlete	-186.37	958.69
HF-HRV recovery (Raw)	Athlete	199.48	1440.83
	Non-athlete	575.02	1346.37
HF-HRV rest (LT)	Athlete	3.07	0.42
	Non-athlete	2.95	0.39
HF-HRV task (LT)	Athlete	3.02	0.40
	Non-athlete	2.83	0.55
HF-HRV post-task (LT)	Athlete	3.08	0.46
	Non-athlete	3.09	0.45
HF-HRV reactivity (LT)	Athlete	-0.01	0.34
	Non-athlete	-0.12	0.52
HF-HRV recovery (LT)	Athlete	0.02	0.40
	Non-athlete	0.18	0.57
VAS unpleasant	Athlete	5.92	2.45
	Non-athlete	7.25	2.32
VAS stress	Athlete	3.55	2.47
	Non-athlete	4.17	2.61
VAS pain	Athlete	5.98	2.82
	Non-athlete	6.74	2.20
RSE	Athlete	0.79	0.54
	Non-athlete	1.02	0.52
HR rest	Athlete	69.26	11.44
	Non-athlete	79.29	11.50
HR task	Athlete	76.09	12.39
	Non-athlete	84.38	16.63
HR post-task	Athlete	67.17	11.22
	Non-athlete	73.94	10.98

*HF-HRV, high frequency heart-rate variability; Raw, HF-HRV variables pre log10 transform; LT, HF-HRV variables post log10 transform; VAS, visual analog scale; RSE, reactivity to social evaluation; HR, heart-rate.*

**Table 2:** Pearson’s r correlations between HF-HRV task and reactivity variables, and VAS scales.

Variable	Unpleasantness	Stress	Pain
HF-HRV task	-0.33^∗^	-0.37^∗∗^	-0.38^∗∗^
HF-HRV reactivity	-0.10	-0.26	-0.17

*^∗^*p* < 0.05. ^∗∗^*p* < 0.01.*

### Gender and Athleticism Differences in HF-HRV

Multivariate tests revealed no significant main effect of gender or athleticism on HF-HRV (see Table 3). However, analysis of each independent variable revealed male participants to have significantly higher levels of resting HF-HRV than female participants, with a medium effect size (see Tables 4, 5). Furthermore, the higher levels of HF-HRV observed in male participants during the SECPT approached significance. No other significant differences were found between the dependent and independent variables.

**Table 3:** Multivariate tests for gender and athleticism on HF-HRV.

Variables	Pillai’s trace	F	*df*	Error *df*	*p*	Partial η^2^
Gender	0.12	2.10	3	47	0.11	0.12
Athleticism	0.02	0.27	3	47	0.85	0.02

**Table 4:** Estimated marginal means.

Dependent			Std.	95% Confidence	
Variable	Group	Mean	error	interval	
				**Lower**	**Upper**
				**bound**	**bound**

HF-HRV rest	Athlete	2.968	0.079	2.809	3.128
	Non-athlete	3.015	0.087	2.840	3.191
	Male	3.119	0.087	2.945	3.293
	Female	2.865	0.080	2.704	3.026
HF-HRV task	Athlete	2.953	0.093	2.766	3.139
	Non-athlete	2.913	0.102	2.708	3.118
	Male	3.072	0.101	2.868	3.275
	Female	2.794	0.094	2.606	2.982
HF-HRV post-task	Athlete	2.984	0.089	2.804	3.164
	Non-athlete	3.075	0.098	2.877	3.273
	Male	3.118	0.098	2.921	3.314
	Female	2.941	0.090	2.760	3.123
HF-HRV reactivity	Athlete	-0.016	0.090	-0.196	0.165
	Non-athlete	-0.103	0.099	-0.301	0.096
	Male	-0.047	0.098	-0.245	0.150
	Female	-0.071	0.091	-0.253	0.111
HF-HRV recovery	Athlete	0.031	0.101	-0.171	0.234
	Non-athlete	0.162	0.111	-0.061	0.385
	Male	0.046	0.110	-0.175	0.268
	Female	0.147	0.102	-0.057	0.352

**Table 5:** Between subjects effects.

Group	HF-HRV			Error
variable	variable	F	*df*	*df*	*p*	Partial η^2^
Gender	Rest	4.64	1	52	0.04	0.09
	Task	4.04	1	52	0.05	0.08
	Post-task	1.76	1	52	0.19	0.04
	Reactivity	0.03	1	52	0.86	0.00
	Recovery	0.46	1	52	0.50	0.01
Athleticism	Rest	0.16	1	52	0.69	0.00
	Task	0.08	1	52	0.77	0.00
	Post-task	0.46	1	52	0.50	0.01
	Reactivity	0.42	1	52	0.52	0.01
	Recovery	0.76	1	52	0.39	0.02

### Validity of Reactivity to Social Evaluation Subscale

Table 6 details the results of the multiple regression analysis. Controlling for gender and athleticism at step one, no significant associations were found between RSE and any of the HF-HRV variables. There were significant associations between RSE and the subjective VAS measures. RSE was significantly correlated with VAS scores for perceived stressfulness and pain, but not unpleasantness.

**Table 6:** Multiple regression analyses for RSE whilst controlling for gender and athleticism at step 1.

				Adjusted
Steps and variables		B	Beta	R^2^	ΔR^2^
Dependent variable: HF-HRV rest					
Step 1	Gender	-0.26	-0.31*	0.07*	
	Athleticism	0.01	-0.02		
Step 2	RSE	0.01	0.02		0.00
Dependent variable: HF-HRV task					
Step 1	Gender	-0.31	-0.32	0.09*	
	Athleticism	-0.06	-0.06		
Step 2	RSE	0.01	0.01		0.00
Dependent variable: HF-HRV post-task					
Step 1	Gender	-0.19	-0.21	0.00	
	Athleticism	0.09	0.10		
Step 2	RSE	0.00	0.00		0.00
Dependent variable: HF-HRV reactivity					
Step 1	Gender	-0.04	-0.04	-0.02	
	Athleticism	-0.10	-0.11		
Step 2	RSE	-0.02	-0.02		0.00
Dependent variable: HF-HRV recovery					
Step 1	Gender	0.10	0.10	0.00	
	Athleticism	0.13	0.13		
Step 2	RSE	0.00	0.00		0.00
Dependent variable: VAS unpleasant					
Step 1	Gender	1.36	0.28*	0.11*	
	Athleticism	0.78	0.16		
Step 2	RSE	1.02	0.22		0.04
Dependent variable: VAS stressfulness					
Step 1	Gender	1.70	0.34*	0.08*	
	Athleticism	-0.06	-0.01		
Step 2	RSE	1.42	.30*		0.08*
Dependent variable: VAS pain					
Step 1	Gender	2.21	0.44**	0.16**	
	Athleticism	-0.13	-0.03		
Step 2	RSE	1.45	0.30*		0.08*

*^∗^*p* < 0.05. ^∗∗^*p* < 0.01.*

## Discussion

In this study, the validity of the PSRS-AA’s RSE subscale was tested in relation to a physiological index of emotion regulation and subjective measures of perceived stress, recorded during a SECPT in student athletes and non-athletes. The validity of the SECPT for inducing stress responses and changes in HF-HRV in student athletes and non-athletes was also tested, along with the effects of gender and athleticism on HF-HRV in the sample population. No significant differences in HF-HRV were observed between the rest and task periods, or the task and post-task periods. However, there were significant relationships between HF-HRV during the SECPT and the subjective VAS measures, with lower levels of HF-HRV being associated with greater perceptions of stress, pain, and unpleasantness.

There was no effect of athleticism on HF-HRV. There was also no overall effect of gender on HF-HRV. However, there was a significant effect on baseline HF-HRV, with males having higher levels than females. This finding is opposed to prior research which has examined gender differences in HF-HRV (Koenig and Thayer, 2016). The effect size observed is similar to that of prior research (Koenig and Thayer, 2016). These findings would suggest that although females exhibit greater HF-HRV than males in the wider population, the opposite is the case among this sample of adolescent students. These findings could be explained by self-selection, with male participants within the sample being more likely to participate if they were capable of coping with multiple sporting and academic commitments compared with other participants from the general, or student, population. The RSE subscale failed to relate to HF-HRV. However, the subscale was significantly associated with the subjective VAS measures of both stress and pain taken immediately after the SECPT.

These results suggest that the SECPT was not effective in producing changes in HF-HRV. However, with lower levels of HF-HRV being associated with greater perceptions of stress and pain, this indicates that the task itself was indeed stressful and painful for participants, and that these perceptions of stress and pain were related to levels of HF-HRV during the SECPT. The SECPT may have failed to produce significant changes in HF-HRV across the three testing periods, due to the demands of the baseline resting and post-task periods. Participants were instructed to remain as still as possible during these periods, which may have been demanding enough to provoke changes in HF-HRV, rather than produce a reliable baseline. Therefore, further research is required to test the validity of the SECPT for measuring changes in HF-HRV in athletic and non-athletic populations.

These results also suggest that there are no differences in HF-HRV between student athletes and non-athletes. This did not support the hypothesis or previous research which has found aerobic fitness to predict higher levels of HF-HRV (Stanley et al., 2013). However, this may be due to the measure of athleticism used in the study (whether participants competed in sporting activities). This did not control for non-athletes with potentially high levels of aerobic fitness. Furthermore, this did not control for students competing in sports at different levels of competition, and the differing levels of aerobic fitness required to compete, in some sports, at higher levels of competition. This is a significant limitation of the present study. Future research may look to conduct more valid and reliable measures of athleticism prior to testing (e.g., training load, measures of cardiovascular fitness) to examine the effect of this variable, and control for its effects, on HF-HRV with athletes. Although there was also no overall main effect of gender on HF-HRV, there was a significant effect on baseline HF-HRV, with males having higher levels than females. This supports the findings of previous research into gender differences in HRV (Woo and Kim, 2015). Therefore, future research into HF-HRV and SR should continue to control for gender differences, particularly when conducting baseline assessments.

With the RSE subscale failing to relate to HF-HRV, this would suggest that the construct of PSR does not directly relate to the physiological processes of stress and emotion regulation. This is despite its relationships with numerous personality traits and psychological outcomes demonstrated in previous research (Britton et al., 2017). On the other hand, this is consistent with previous research which has found no association between physiological measures of reactivity (such as heart rate and respiratory sinus arrhythmia) and perceptions of reactivity in adolescent participants (Evans et al., 2013). Previous research has, however, found associations between neuroendocrine indices and perceived reactivity (Schlotz et al., 2011b; Evans et al., 2013), suggesting that measures such as the PSRS-AA may be more reliable alternatives to these indices, rather than physiological measures.

With perceived reactivity relating to the subjective VAS measures of both stress and pain taken immediately after the SECPT, this suggests that PSR relates more strongly to perceptions of stress rather than physiological processes. This is consistent with Lazarus and Folkman’s transactional model of stress, which proposes that stress emerges from an interaction between an individual and their environment, plus the subjective appraisal of potentially stressful events.

### Implications

These findings have several implications for future research. The relationship between PSR and emotion regulation requires further investigation. With PSRS-AA scores not being associated with HF-HRV, but perceived stress in response to the SECPT, future research may wish to avoid using the PSRS-AA as a replacement for physiological measures of SR. Instead, the PSRS-AA may be used as an alternative or complementary measure, which more closely aligns with cognitive theories and processes of stress (i.e., Lazarus and Folkman, 1987), rather than physiological processes. However, future research may wish to test the validity of the PSRS-AA further in relation to other physiological or neuroendocrine markers of SR. HF-HRV is a marker of para-sympathetic activation, therefore future research could conduct the SECPT with measures of sympathetic reactivity (e.g., salivary alpha amylase or skin conductance response). Furthermore, different lab procedures could also be used to provoke stress responses, given that there were no significant differences in HF-HRV between the different phases of the SECPT (e.g., The Trier Social Stress Test; Kirschbaum et al., 1993). Independent from the findings related to the PSRS-AA, male participants were found to have higher levels of HF-HRV at rest than females. Future research should therefore continue to control for gender differences when examining HF-HRV, in both student athletes and non-athletes.

In terms of future applied practice, these findings also have some implications. Results from the SECPT would suggest that adolescent athletes scoring high on the PSRS-AA should be prioritized for interventions which address the cognitive appraisal of stress under conditions of social evaluation. Given the lack of a relationship between the PSRS-AA and HF-HRV, interventions designed to directly address physiological processes (i.e., relaxation techniques) may not be effective for young athletes scoring highly on the scale. Overall, given that adolescent athletes regularly face stressors associated with social evaluation, the PSRS-AA can be utilized to identify, and thus support, young athletes at greatest risk of experiencing decreased satisfaction with their sporting experience and thus dropout.

### Limitations

The present study used a log10 transform to analyze the HF-HRV, as it was not normally distributed. Recent recommendations have suggested that natural log transformations are more intuitive and more appropriate for analyzing HF-HRV data and allow for the possibility of comparing data between studies (Laborde et al., 2017). Therefore, the log10 transformed data within the present study makes the findings difficult to compare with previous research.

As previously discussed, the use of mere participation in competitive sport as a measure of athleticism can be seen as a significant limitation of the present study. This binary categorization was used in order to control for effect of athleticism on HF-HRV (Stanley et al., 2013) as it was a less time-consuming method than more detailed assessments of athleticism or cardiovascular fitness. A more valid measure may have yielded significant effects for athleticism on HF-HRV, and so future studies would be advised to consider more comprehensive assessments of this variable (e.g., competition level, weekly hours of practice and competition).

## Conclusion

This study refines the validity of the PSRS-AA for assessing individual differences in adolescent athletes’ PSR. Little support was obtained regarding the relationship between PSR and psycho-physiological indices of emotion regulation. However, perceptions of stress, pain and unpleasantness in response to a novel stressor were associated with greater PSR, building upon the PSRS-AA’s criterion validity. Future research should look to examine the relationship between SR and emotion regulation further and consider individual differences in PSR when exploring stress appraisals and subjective processes within stress-coping and emotion regulation. Overall, this further supports PSR as a significant individual difference affecting the experience of stress in adolescent athletes that is worthy of further research.

## Ethics Statement

This study was carried out in accordance with the recommendations of “Bournemouth University Human Ethics Committee” with written informed consent from all subjects. All subjects gave written informed consent in accordance with the Declaration of Helsinki. The protocol was approved by the “Bournemouth University Human Ethics Committee.” For all participants under the age of 16, parents or guardians were also sent an information sheet and asked to provide written consent.

## Author Contributions

DB, EK, and RP conceived and designed the study, experiments, edited and critically revised the manuscript, and approved the final version of the manuscript. DB analyzed the data, drafted the manuscript, and prepared table/figures. DB and RP interpreted results of research.

## Conflict of Interest Statement

The authors declare that the research was conducted in the absence of any commercial or financial relationships that could be construed as a potential conflict of interest.
